# Evaluation of the pulmonary vein ostia during the cardiac cycle using electrocardiography-gated cardiac computed tomography in cats

**DOI:** 10.3389/fvets.2022.1013963

**Published:** 2022-09-27

**Authors:** Junyoung Kim, Dae-Hyun Kim, Kitae Kim, Dayoung Oh, Jihye Choi, Junghee Yoon

**Affiliations:** ^1^Medical Imaging, Helix Animal Medical Center, Seoul, South Korea; ^2^College of Veterinary Medicine and the Research Institute for Veterinary Science, Seoul National University, Seoul, South Korea; ^3^College of Veterinary Medicine, Chungnam National University, Daejeon, South Korea; ^4^Medical Imaging, BIEN Animal Medical Center, Bucheon, South Korea

**Keywords:** caudodorsal ostium, feline, hypertrophic cardiomyopathy, left atrium to aorta ratio, left cranial ostium, right cranial ostium

## Abstract

Several studies in humans have provided detailed descriptions of the anatomy of the pulmonary veins (PVs) and their ostia for the implementation of thoracic interventions, such as radiofrequency ablation, for patients with atrial fibrillation. These studies have shown that electrocardiography (ECG)-gated multidetector computed tomography (MDCT) can evaluate the dimensional variations in the PVs or ostium according to the cardiac cycle. However, few studies have examined the PVs or ostia using MDCT in veterinary medicine. Therefore, this study investigated the variation in the diameter of the PV ostium in cats during the cardiac cycle using ECG-gated MDCT and determined the correlation between the size of the heart or left atrium (LA) and diameter of the PV ostium. This study included six cats, including five normal animals and one cat with hypertrophic cardiomyopathy. The PVs were found to drain into the LA *via* three ostia, i.e., the right cranial ostium, left cranial ostium, and caudodorsal ostium. Moreover, a diametric variation was observed in all PV ostia according to the cardiac cycle phase on ECG-gated MDCT: the maximal diameter was observed at the end of ventricular systole, and the minimal diameter was observed at the end of ventricular diastole for each PV ostium. There were no significant correlations between the heart or LA size and maximal or minimal diameter of each of the three PV ostia (*p* > 0.05); however, the enlargement of each PV ostium at the end of ventricular systole differed significantly from that at the end of ventricular diastole. This study suggested the clinical feasibility of ECG-gated MDCT in providing more detailed anatomical information about the PVs, including the dimensional changes during the cardiac cycle in cats. Based on this study, knowledge of the variations in the PV ostium offers interesting avenues for research into the effect of PV function. Furthermore, ECG-gated MDCT could allow for greater clinical application of interventional procedures in animals with various cardiac diseases.

## Introduction

The pulmonary veins (PVs) and their ostia, which are important sources of ectopic atrial activity, have been implicated in chronic and paroxysmal atrial fibrillation (1–4). Radiofrequency ablation of the PV is performed for patients with atrial fibrillation to disconnect the PV electrically from the left atrium (LA) in humans; thus, detailed anatomical information about the PV is important for catheter size selection during the ablation procedure (1–3, 5). The PV also constitutes an essential aspect of thoracic interventions, such as lung transplantation and pneumonectomy; moreover, pulmonary venous congestion is a clinically important indicator of elevated pulmonary venous pressure, a cause of pulmonary edema (6, 7). Therefore, detailed anatomical knowledge of the PV is important clinically in both human and veterinary medicine.

Technological advancement and electrocardiographic (ECG) gating have enabled detailed visualization of the anatomical features of cardiovascular structures using multidetector computed tomography (MDCT) (1, 2). Several studies in humans have reported detailed anatomical information on the PV and ostium, and ECG-gated MDCT was used to evaluate the dimensional variations of the PV or ostium according to the cardiac cycle (1, 2, 6, 8, 9). These studies in human medicine have demonstrated significant dimensional differences in the PV and ostia between the ventricular end-systole and ventricular end-diastole, which apparently become less significant further from the LA (2, 9). Moreover, patients with chronic atrial fibrillation and left atrial enlargement may have larger PVs and ostia than those with paroxysmal atrial fibrillation and a normal-sized atrium (1, 4). These results suggest the clinical significance of ECG-gated MDCT in evaluating the PV and ostium in human medicine.

However, few studies have evaluated the PV or ostium using MDCT in veterinary medicine. Most studies of pulmonary vessels using MDCT have focused primarily on the pulmonary arteries, and studies related to the PVs have reported only on pulmonary venous drainage patterns and the number of PV ostia in dogs and cats (10–15). To the best of our knowledge, no study has reported the diametric variation in the PV or ostium during the cardiac cycle using ECG-gated MDCT in veterinary medicine. Additionally, although the PV to pulmonary artery (PA) ratio has been evaluated as a predictive factor for congestive heart failure using echocardiography, evaluation of the PV or ostium using MDCT may be clinically useful in the future, considering the limitations of echocardiographic examination arising from its great dependence on the operator's scan techniques and/or the patient's respiration.

Therefore, this study aimed to investigate the variation in the diameter of the PV ostium in cats during the cardiac cycle using ECG-gated MDCT, as well as the correlation between the size of the heart or LA and diameter of the PV ostium.

## Materials and methods

### Population

This study represents a retrospective analysis of a subset of the original, prospective study. Data from five clinically normal cats and one cat with hypertrophic cardiomyopathy (HCM) that underwent ECG-gated MDCT were analyzed retrospectively. The study design and care, as well as animal maintenance, followed protocols approved by the institutional animal care and use committee of Seoul National University in February 2022 (approval number: SNU-220113-4). Medical history and informed consent were obtained from the owners for all client-owned cats prior to all procedures. Six domestic short-haired cats with no clinical signs provided by the owners were included. Before MDCT examination, all cats underwent basic health tests, including physical examination, complete blood counts, serum biochemistry, and electrolyte tests. Cardiac evaluation was performed using N-terminal pro-B-type natriuretic peptide (NT-proBNP) testing, thoracic radiography, and transthoracic echocardiography (Aplio 500, Toshiba, Canon Medical Systems Co., Otawara, Japan). Two-dimensional, M-mode, and Doppler echocardiography was performed for all cats. The time interval between all basic health tests and MDCT examination for each cat was <5 days.

### Anesthesia

An intravenous 24-G catheter was placed in the right cephalic vein for premedication and injection of the contrast agent during MDCT. General anesthesia was induced as follows: premedication with butorphanol (0.2 mg/kg intravenously; 1 mg/mL, Butophan^®^ Myungmoon Pharm Co., Ltd., Seoul, Republic of Korea), induction with propofol (6 mg/kg intravenously; 10 mg/mL, Provive^®^ 1%, Myungmoon Pharm Co., Ltd.,), and maintenance with isoflurane (Isotroy^®^ 100, Troikaa Pharm Ltd., Gujarat, India) in a gaseous mixture of 100% oxygen in air *via* an endotracheal tube. End-tidal carbon dioxide levels were maintained between 35 and 45 mmHg using a mechanical ventilator. During anesthesia, the heart rate, oxygen saturation, and end-tidal carbon dioxide were monitored continuously using ECG and pulse oximetry. Data acquisition was initiated within 5–10 min after anesthesia induction to ensure the stability of anesthetic conditions. For individual scans, apnea was induced by breath-holding at inspiration immediately before the scan. All cats were monitored until recovery from anesthesia.

### ECG-gated MDCT

All MDCT examinations were performed using an 80-row, 160-multislice CT system (Aquilion Lightning, Canon Medical Systems Co., Otawara, Japan). During the examination, the patients were positioned in sternal recumbency on a CT table with the neck extended and the forelimbs placed caudally. ECG leads were attached to the paws; ECG data were recorded simultaneously during spiral MDCT examination. The scan protocol was as follows: voltage, 120 kVp (kVp: kilovoltage peak); gantry speed, 0.5 s/rotation; slice collimation, 0.5 mm × 80; 150 mA; slice thickness, 0.5 mm; and pitch factor, 0.813. All patients underwent a non-ECG-gated scan, followed by a retrospective ECG-gated scan after a short interval (5 min) to allow washout of the contrast medium from the heart. All cats underwent a pre-contrast MDCT scan of the full thorax from the thoracic inlet to the most caudal border of the lungs before the post-contrast studies. For non-ECG-gated scans, the following were injected into the cephalic vein using a dual power injector (OptiVantageTM DH, Mallinckrodt, Dublin, Ireland) at a rate of 1.5 mL/s: a biphasic injection, a non-ionic contrast medium (300 mgI/mL, Omnipaque; GE healthcare, Seoul, Republic of Korea) of 1.5 mL/kg, followed by a saline flush of 1.5 mL/kg. After 8 s of contrast injection, five sequential scans were performed over the cardiac silhouette from the cranial to the caudal side at 5 s intervals. The delay time for retrospective ECG-gated scans was 14 s in all cats; this was determined based on non-ECG-gated sequential scan images without bolus tracking to reduce radiation exposure and anesthesia time. The contrast medium administration for ECG-gated MDCT was conducted in the same manner as that for the non-ECG-gated scan. For data postprocessing, images were reconstructed in multiple datasets, by increasing the temporal reconstruction window in 10% increments within the cardiac cycle, centered over the 0–90% R-R interval. All images were reviewed by three veterinary diagnostic imaging experts on a dedicated viewing station using specialized software (Vitrea 7.12, Vital Images, Minnetonka, MN, USA), which depicted maximum intensity projection (MIP), three-dimensional volume rendering, and multiplanar reconstructions to optimize visualization of the PV and ostium.

### Evaluation of the PV ostium in cats

#### Number of the PV ostia within the LA and classification of the pulmonary venous drainage system just before opening into the PV ostium

The pulmonary venous drainage system was classified according to a previous study (10): (a) separate, when the PVs drained independently into the LA; (b) short common trunk, when two or more PVs fused by forming a “short neck” just before opening into the LA; or (c) long common trunk, when two or more PVs fused by forming a “long neck” just before opening into the LA.

#### Variation in the PV ostial diameter during the cardiac cycle on ECG-gated MDCT

**Figure 1:**
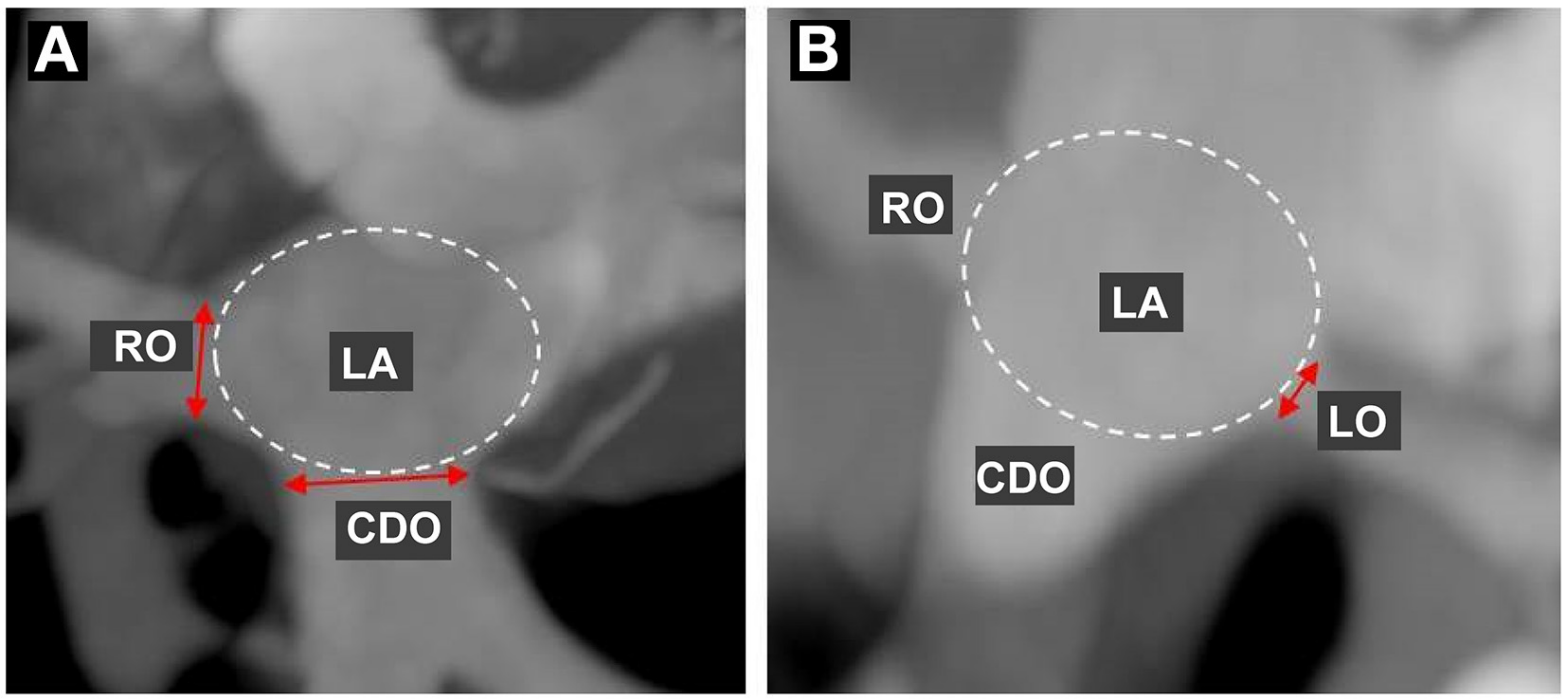
Measurement of the pulmonary vein (PV) ostial diameter. First, the maximum intensity projection (MIP) of the oblique transverse or coronal planes for best visualization of each PV ostium were selected using multiplanar reconstruction, and then each PV ostial diameter during the cardiac cycle was measured and compared at the same cross-section. Each ostium is defined as the point of inflection between the PV wall and the LA wall, and the ostial diameter was measured from the PV wall to the PV wall. **(A)** indicates the measurement of the diameter in the right cranial ostium (RO) and caudodorsal ostium (CDO), and **(B)** indicates the left cranial ostium (LO).

In 10 sets of phase reconstruction data, ranging from 0 to 90% in 10% increments of the R-R interval on the ECG in all cats, the diameter of each PV ostium entering the LA according to the cardiac cycle was measured and compared through the same cross-section at the MIP oblique transverse or coronal planes, by dropping a perpendicular to the long axis of the PV. The ostium is defined as the point of inflection between the PV and LA walls, and the ostial diameter was measured from PV wall to PV wall (Figure 1). Measurement of each PV ostial diameter according to the cardiac cycle was performed three times each by three veterinary diagnostic imaging experts (J Kim, K Kim, and D Oh), and the average value for the average value of three veterinary diagnostic imaging experts was determined as the final value. The diameter of each PV ostium at ventricular end-systole (30–40% R-R interval) and ventricular end-diastole (0%, 70–90% R-R interval) was selected and compared among all cats. Furthermore, the reconstruction windows showing the maximal and minimal diameters of the ostia of all PV measured during the cardiac cycle were selected.

#### Correlation between the PV ostial diameter and sizes of the heart or LA

In all cats, the maximal or minimal values of the PV ostial diameters were compared with the vertebral heart score (VHS) on thoracic radiography or LA to aorta (AO) ratio on echocardiography.

### Statistical analyses

All data were expressed as the mean ± standard deviation. Statistical analyses were performed using IBM SPSS 26.0 (IBM Corp., Armonk, NY, USA). As the data were not normally distributed owing to the small sample size, we used non-parametric tests, except for the comparison of the PV ostial diameter between the end-systolic and end-diastolic phases. Spearman's rho test was used to identify the statistical correlations between age, body weight (BW), VHS or LA/AO ratio, and maximal or minimal diameter of each PV ostium. The Kruskal-Wallis test was used to compare all values with respect to sex. An independent *t*-test was used to compare the mean value of each PV ostium at the ventricular end-systolic and ventricular end-diastolic phases. A one-sample *t*-test was used to compare the statistically significant differences among the five clinically normal cats and one cat with HCM for the BW, VHS, LA/AO ratio, and maximal/minimal diameter of each PV ostium. A *p*-value < 0.05 was considered statistically significant.

## Results

Six domestic short-haired cats in this study included three spayed female and three male (one intact, two neutered). The mean age was 4.85 ± 3.74 years, the mean BW was 4.82 ± 1.0 kg, the mean VHS was 7.03 ± 0.72 v, and the mean LA/AO was 1.27 ± 0.22 (Table 1). Of the six cats, five were normal in all basic health tests, although one HCM cat (case 6) showed regional thickening (0.66 cm) of the interventricular septum and a positive result in the NT-ProBNP test (Table 1). One HCM patient had a systemic blood pressure of 150 MmHg and a normal serum thyroid hormone concentration at 2.1 μg/dL (reference range, 0.6–3.9 μg/dL).

**Table 1:** Results of the signalment, vertebral heart scale (VHS), left atrium (LA) to aorta (AO) ratio, and N-terminal pro-B-type natriuretic peptide (NT-proBNP) in six domestic short hair cats.

**Case No**.	**Age (years)**	**Sex**	**Body weight (kg)**	**VHS**	**LA/AO**	**NT-proBNP**
1	3	Spayed female	4.14	7.2 v	1.44	Negative
2	3.6	Intact male	4.24	6.0 v	1.37	Negative
3	1	Spayed female	4.4	7.2 v	1.11	Negative
4	2.7	Spayed female	4.48	6.9 v	1.0	Negative
5	8	Castrated male	6.8	6.7 v	1.13	Negative
6	10.8	Castrated male	4.85	8.2 v	1.56	Positive

**Figure 2:**
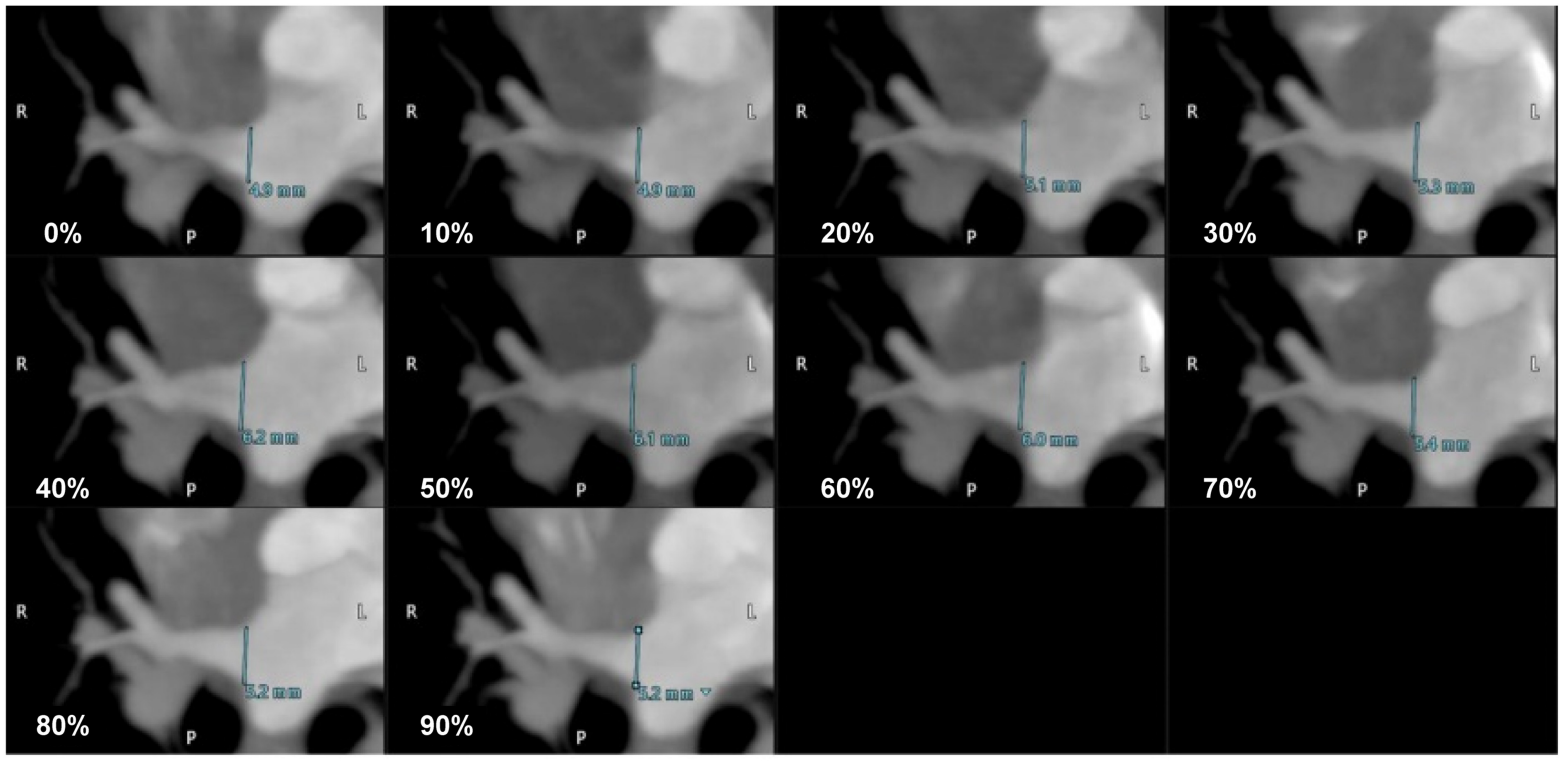
The maximum intensity projection (MIP) oblique transverse images showing the dimensional variation of the right cranial ostium (RO) according to the cardiac cycle (0–90% R-R interval) in a cat.

**Figure 3:**
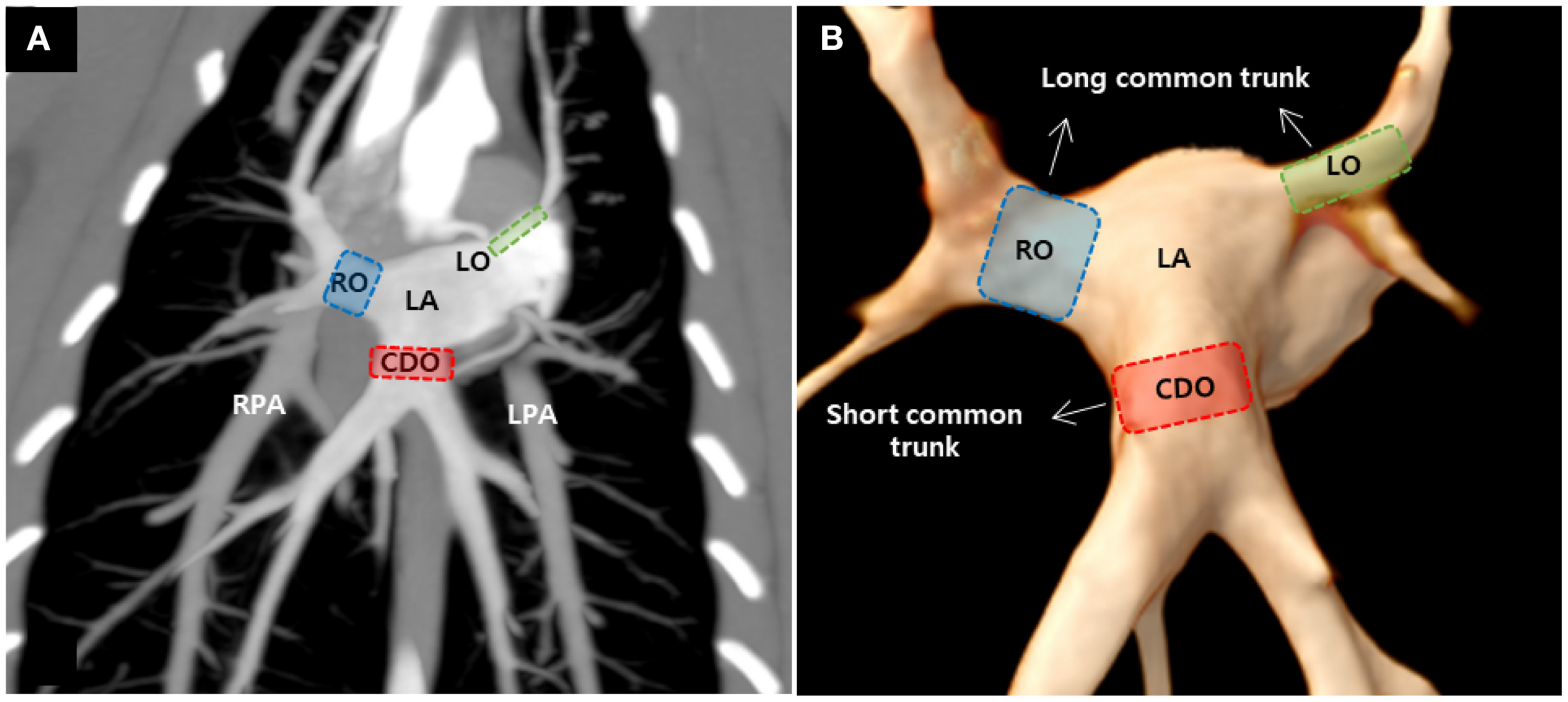
Maximum intensity projection **(A)** and three-dimensional volume rendered images **(B)** showing the pulmonary veins (PVs) ostia entering the left atrium (LA) in cats. There were three PV ostia, consisting of the right cranial ostium (RO), left cranial ostium (LO), and caudodorsal ostium (CDO), in this study. RPA, right pulmonary artery; LPA, left pulmonary artery.

ECG-gated MDCT scan allowed the measurement of the ostial diameter of each PV entering the LA during the cardiac cycle in all cats (Figure 2). The total time from induction of anesthesia to the end of MDCT examination ranged between 25 and 35 min, with an average of 30 min per animal. The heart rate during the MDCT scan ranged from 120 to 150 bpm in all cats. No complication associated with the anesthetic protocol was documented during the procedure. The PVs drained into the LA *via* three ostia, i.e., the right cranial ostium (RO), left cranial ostium (LO), and caudodorsal ostium (CDO), which were identified in all cats (Figure 3) (10). The RO, draining from the PVs of the right cranial and middle lung lobes, formed a long common trunk before opening into the right cranial part of the LA. The LO, draining from the PVs of the cranial and caudal parts of the left cranial lung lobe, also formed a long common trunk before opening into the left cranial part of the LA. The CDO, draining from the PVs of the bilateral caudal and accessory lung lobes, formed a short common trunk before opening into the caudodorsal part of the LA. These findings were similar to those of a previous study (10).

**Figure 4:**
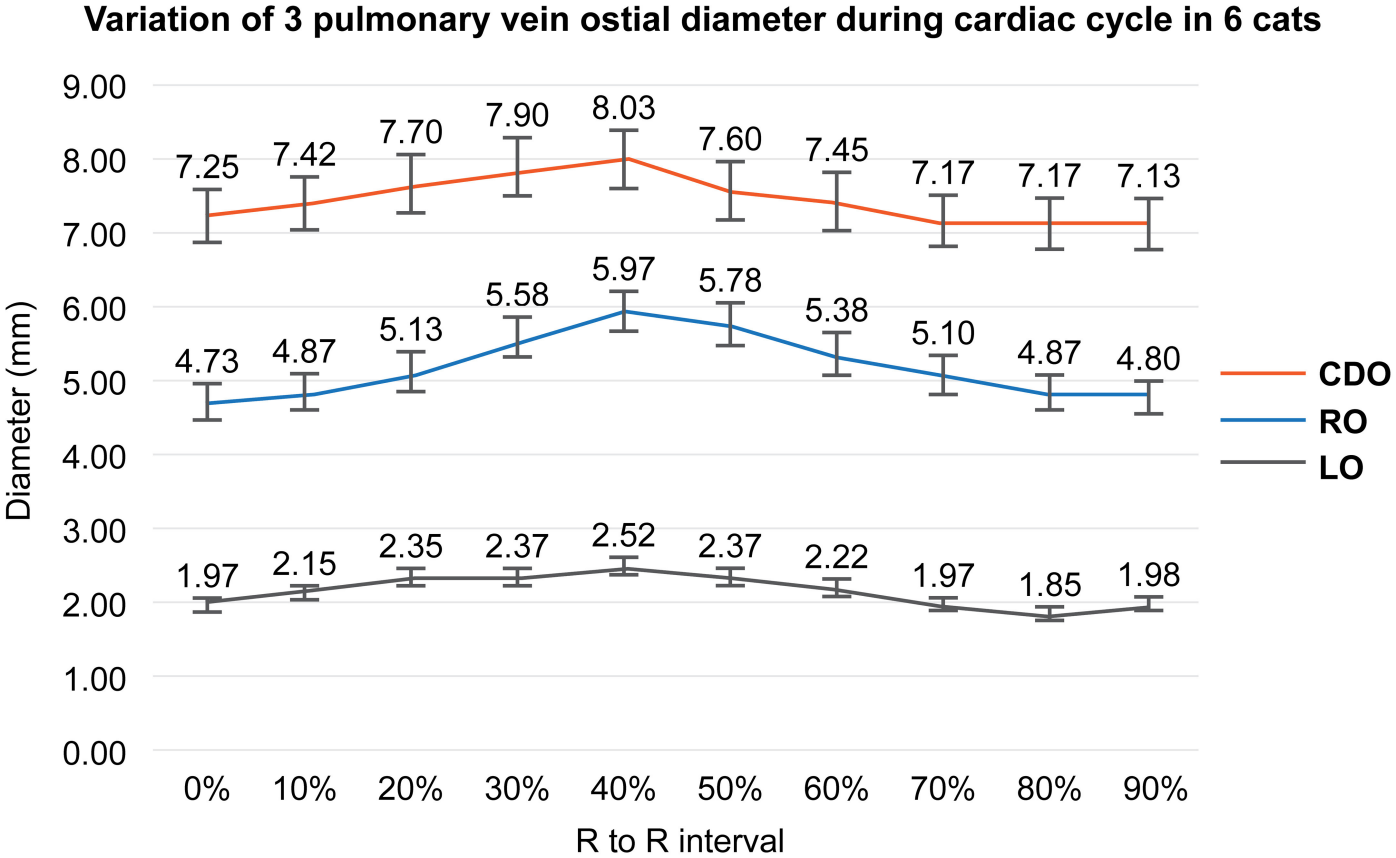
Variation of each pulmonary vein (PV) ostial diameter during the cardiac cycle in this study. This figure shows the mean value of each PV ostial diameter according to the cardiac cycle in six cats. All PV ostia show the maximum at the ventricular end-systole (40% R-R interval) and minimum at the ventricular end-diastole (0%, 90% R-R interval). RO; right cranial ostium, CDO; caudodorsal ostium, LO; left cranial ostium.

As a result of PV diameter measurement using a specialized software, the average value of three veterinary diagnostic imaging experts showed consistent results. In this study, the CDO was found to be the largest and the LO the smallest, and all ostia showed the dimensional variation during the cardiac cycle (Table 2). The maximal diameter showed at the end-systole (30–40% R-R interval) except in two cases (50% R-R interval) and the minimal at the end-diastole (0%, 70–90% R-R interval) except in one case (50% R-R interval) (Table 2). In six cats, the average diameter of each PV ostium according to the cardiac cycle was the maximum at the end-systole and minimum at the end-diastole (Figure 4). There was a significant difference in the mean diameter of each PV ostium between the end-systole and end-diastole (*p* < 0.05) (Table 3). There were no statistical correlations between the maximal or minimal diameter of all PV ostia and age, BW, sex, VHS, or LA/AO ratio (*p* > 0.05). One HCM cat showed a significant enlargement in the VHS and LA/AO ratio compared with the other five normal cats (*p* < 0.05), but showed no significant differences in all PV ostial diameters between the two groups (*p* > 0.05).

**Table 2:** The mean value of the three pulmonary vein ostial diameters according to the cardiac cycle in six cats.

**RR interval**	**Case 1**			**Case 2**			**Case 3**			**Case 4**			**Case 5**			**Case 6**		
	**RO**	**CDO**	**LO**	**RO**	**CDO**	**LO**	**RO**	**CDO**	**LO**	**RO**	**CDO**	**LO**	**RO**	**CDO**	**LO**	**RO**	**CDO**	**LO**
0%	4.2	7.4	1.8	4.9	7.4	1.7	4.5	7.2	2.0	4.8	7.1	1.9	4.8	7.1	2.0	5.2	7.3	2.4
10%	4.6	7.6	2.0	4.9	7.3	1.8	4.5	7.4	2.2	4.9	7.5	2.0	4.9	7.4	1.9	5.4	7.3	3.0
20%	4.7	7.8	2.5	5.1	7.4	2.0	4.5	7.6	2.2	5.3	7.8	2.2	5.4	7.4	2.3	5.8	8.2	2.9
30%	5.1	7.9	2.4	5.3	7.7	1.9	5.5	8.1	2.2	5.7	7.9	2.5	5.6	7.5	2.2	6.3	8.3	3.0
40%	4.9	8.4	2.2	6.2	7.7	2.1	5.4	8.0	2.3	6.8	7.7	2.6	6.1	8.3	2.5	6.4	8.1	3.4
50%	4.7	7.7	2.0	6.1	7.5	2.2	5.4	7.9	2.5	6.6	7.0	2.5	5.8	7.7	2.4	6.1	7.8	2.6
60%	4.4	7.8	2.0	6.0	7.5	2.0	5.4	8.1	2.2	5.4	6.8	2.0	5.3	7.0	2.1	5.8	7.5	3.0
70%	4.5	7.7	2.0	5.4	7.5	1.7	5.3	7.1	2.2	5.2	6.9	1.6	5.2	6.5	1.9	5.0	7.3	2.4
80%	3.9	7.7	1.8	5.1	7.2	1.6	5.0	6.7	1.8	4.9	7.3	1.8	5.0	6.8	1.7	5.3	7.3	2.4
90%	4.4	7.5	1.8	5.2	7.2	1.5	4.5	6.7	2.0	4.6	7.3	1.8	4.6	6.6	1.9	5.5	7.5	2.9

All values are indicated in mm. RO, right cranial ostium; CDO, caudodorsal ostium; LO, left cranial ostium.

**Table 3:** The comparison of the mean PV ostial diameter between the ventricular end-systole (30–40% R-R interval) and ventricular end-diastole (0%, 70–90% R-R interval) in six cats.

	**End-diastole (mm)**	**End-systole (mm)**	***p-*value**
RO	4.875 ± 0.404593	5.775 ± 0.580165	0.000
CDO	7.17917 ± 0.330979	7.96667 ± 0.283912	0.000
LO	1.94167 ± 0.320213	2.44167 ± 0.412219	0.000

All values are expressed as means ± standard deviations. There is a significant difference between the ventricular end-systole and ventricular end-diastole in all PV ostia (p < 0.05). PV, pulmonary vein; RO, right cranial ostium; CDO, caudodorsal ostium; LO, left cranial ostium.

## Discussion

This study showed that the diameter variation of all PV ostia according to the cardiac cycle in cats could be identified on ECG-gated MDCT. As in humans, the maximal diameter of each PV ostium corresponded to the phase of the end of ventricular systole, and the minimal diameter was measured in the end of ventricular diastole. Furthermore, this study showed significant differences in each of the three PV ostia enlarged at the end of ventricular systole compared with those at the end of ventricular diastole. In humans, the right superior PV ostium was found to be the largest and the right inferior PV ostium was the smallest, with the greatest dimensional change in the superior PV compared with the inferior PV, and the left superior PV exhibiting the greatest change (2). However, in our feline study, the diameter of the CDO was the largest and that of the LO was the smallest. In addition, the diameter change at the RO and CDO was larger than that of the LO. These anatomical differences suggest that further extensive studies are needed to determine the difference between human and cats in the effect of PV function as well as the pathophysiology of atrial fibrillation, associated with the extent and degree of myocardial sleeve at each PV ostium, and pulmonary congestion or edema.

Pulmonary venous blood flow is typically biphasic: the first phase of flow occurs during ventricular systole, and the second phase of flow occurs during ventricular diastole (2, 9). The PV orifice area also changes considerably during the cardiac cycle (2). During ventricular systole, blood flows from the PVs into the LA upon closure of the mitral valve. This is driven by left ventricular long-axis shortening, which lengthens the LA, thus increasing the pressure gradient between the PV and LA, and in effect, “sucks” blood into the LA (2). During early diastole, while blood is flowing into the left ventricular chamber, there is a drop in LA pressure and blood is passively pulled into the LA, as it moves through the mitral valve into the left ventricular chamber. By mid to end ventricular diastole, the pulmonary venous pressure equalizes with ventricular diastolic pressure, because of which antegrade pulmonary venous flow begins to cease.

In small animal clinics, clinicians routinely evaluate the relative size of the PV and PA on radiographs by comparing them to the size of the ribs (16). However, radiographic examination may vary depending on the breed, age, obesity, and underlying thoracic disease. In addition, the PV to PA ratio using echocardiography has been suggested as a predictive factor for discriminating healthy or subclinical patients with cardiomyopathy and patients with congestive heart failure in dogs and cats (7, 16, 17). In a feline study, healthy and subclinical cats did not differ in the PV to PA ratio in the echocardiography; meanwhile, cats with congestive heart failure had a larger ratio compared with healthy and subclinical cats (17). In accordance with a previous study, our data also showed that the PV ostial diameter in one subclinical HCM cat did not differ from that in the five normal cats, although the maximal diameter in the CDO and LO of one HCM cat was slightly larger than those of the five normal cats. However, considering several limitations in the echocardiographic examination, highly dependent on the operator's technique, obesity and patient's heart rate or respiration, the evaluation of all PV ostia using ECG-gated MDCT could be valuable and useful in veterinary clinics in the future. There is an additional limitation which is that the echocardiography can image only in the RO region and not all three PV ostia.

In this study, the pulmonary venous drainage patterns of six cats showed three PV ostia, unlike that in humans who have four PV ostia, similar to a previous feline study (10). In humans, many studies have shown that the superior veins have longer myocardial sleeves than the inferior veins, with the left superior PV having the longest sleeve and the right inferior PV having the shortest (2). In this study, in all cats, the RO and LO had a long common trunk, and the CDO had a short common trunk. Although further studies with histological examination should be accompanied, we suggest the possibility that myocardial sleeves of the RO and LO are longer than that of the CDO and could be considered as a pathophysiology of atrial fibrillation.

Although studies in humans have reported that patients with an enlarged LA may have larger PVs than those with a normal-sized LA and the diameter of the left superior PV was significantly larger in men than in women, there was no significant correlation between the heart or LA size, sex, BW, age, and the PV ostial diameter in this feline study (1, 4). This may be attributed to a slightly larger LA/AO ratio (1.56) in one HCM patient than in the five normal cats and six cats showing normal mitral E flow on echocardiography. Therefore, although there was no statistical significance, further extensive research may be necessary for many populations with various degrees of LA/AO ratio or high mitral E flow. With these future studies, it may be clinically useful to establish new criteria for an early prediction or cut-off value of pulmonary congestion or pulmonary edema in patients with early stages of the various heart diseases.

As aforementioned, one limitation of our study was the small population. Other limitations include the possibility of inaccurate PV ostial diameter measurement on MDCT as well as no confirmation by biopsy or necropsy. Although our data could be sufficiently reliable in that we compared over the same cross-section and showed significant differences in all PV ostia between the ventricular end-systole and ventricular end-diastole, further research is needed to measure the cross-sectional area using three-dimensional imaging or vessel tracking in all PV ostia, because the diameter of each PV ostium by two-dimensional measurement is more susceptible to inaccuracies by shifting the imaging plane and choice of window width and level settings for display of the CT angiographic data which can affect the measured vein diameter and cross sectional area. In addition, the volume measurement of each PV common trunk may be useful as an additional predictive factor for the pulmonary congestion in the future. Finally, the risk of anesthesia can be significant, especially in animals with heart disease. Thus, difficulties in practical application of MDCT imaging may also be considered.

Despite some limitations and the necessity of further research, this study suggests the clinical feasibility of using ECG-gated MDCT to provide more detailed anatomical information on the PV, including the dimensional changes during the cardiac cycle in cats, similar to that in humans. ECG-gated MDCT of the PVs may also be useful, because the non-invasive, easily reproducible nature and ability to demonstrate three-dimensional anatomy are worthwhile advantages over other techniques. Based on this study, knowledge of variation in the PV ostium offers interesting avenues for potential research into the effect of PV function in felines with atrial fibrillation and early detection or variable distribution of pulmonary congestion or edema in cats. Furthermore, ECG-gated MDCT could allow for greater clinical application of interventional procedures for various cardiac diseases, including radiofrequency ablation, for the treatment of atrial fibrillation in veterinary clinics.

## Data availability statement

The original contributions presented in the study are included in the article/supplementary files, further inquiries can be directed to the corresponding author.

## Ethics statement

The animal study was reviewed and approved by the study design and care, as well as animal maintenance, followed protocols approved by the institutional animal care and use Committee of Seoul National University (Approval Number: SNU-220113-4). Written informed consent was obtained from the owners for the participation of their animals in this study.

## Author contributions

JK: setting the direction and design for the overall study and writing the main paper. D-HK: writing the thesis, analyzing and interpreting data, and reviewing the patients' conditions. KK: collecting and analyzing the patients' data and contributing to the direction of this study. DO: analyzing data and considering clinical aspects of the study. JC: additional data analysis and interpretation, revising paper, and great advice and assistance in the final approval of this study. JY: coordinating the overall flow and direction of the study and writing and editing the paper. All authors contributed to the article and approved the submitted version.

## Conflict of interest

Author JK was employed by company Helix Animal Medical Center. Author KK employed by company BIEN Animan Medical Center. The remaining authors declare that the research was conducted in the absence of any commercial or financial relationships that could be construed as a potential conflict of interest.

## Publisher's note

All claims expressed in this article are solely those of the authors and do not necessarily represent those of their affiliated organizations, or those of the publisher, the editors and the reviewers. Any product that may be evaluated in this article, or claim that may be made by its manufacturer, is not guaranteed or endorsed by the publisher.

## Abbreviations

AO: aorta
BW: body weight
CDO: caudodorsal ostium
ECG: electrocardiography
HCM: hypertrophic cardiomyopathy
LA: left atrium
LO: left cranial ostium
MDCT: multidetector computed tomography
MIP: maximum intensity projection
NT-proBNP: N-terminal pro-B-type natriuretic peptide
PV: pulmonary vein
RO: right cranial ostium
VHS: vertebral heart score.
